# Heterotopic ossification in primary total hip arthroplasty: risk factor analysis

**DOI:** 10.1007/s00590-022-03244-9

**Published:** 2022-03-19

**Authors:** Alessandro Aprato, Simone Cambursano, Stefano Artiaco, Stefano Bevilacqua, Paolo Catalani, Alessandro Massè

**Affiliations:** University of Turin, Viale 25 aprile 137 int 6, 10133 Turin, Italy

**Keywords:** Total hip arthroplasty, Heterotopic ossification, Brooker classification, Hip arthroplasty outcomes

## Abstract

**Background:**

Aim is to identify if age, sex, type of posterolateral approach (mini vs standard), surgical time and time from surgery to drainage removal were independent risk factors for heterotopic ossifications after total hip arthroplasty.

**Materials and methods:**

Patients who underwent a THA with posterolateral approach during a 15 years period were included. The exclusion criteria were absence of X-rays follow-up or HO prophylaxis protocol adoption. The following data were collected: age, sex, type of approach (classical/minimal-invasive), surgical time, time from surgery to drainage removal. Two orthopedic surgeons independently reviewed the 2 years follow-up X-rays and classified the HO according to Brooker classification. Severe HO was defined if HO were classified as major than grade 2. Correlation between severe HO and risk factor has been tested with multivariable analysis.

**Results:**

About 1225 patients were included: mean age of 63.8 years, 504 were men. HO were found in 67.6%. Men showed higher severe HO rate than woman (44.1% vs 29.1%, *p* = 0.001). Patients older than 65 years showed higher severe HO rate (30.3% vs 39.9%, *p* = 0.002). Standard posterolateral approach was performed in 75.4% and severe HO rate was 32.8% versus 27.1% in those treated with the minimally invasive approach (*p* = 0.067).

In 75.6% of cases surgery lasted less than 90 min and this group showed a severe HO rate in 29.1%, while patient with longer surgical time showed a rate of 35.7% (*p* = 0.033). In 47.4% of patients, the drainage was removed in the first post-operative day, in this group severe HO rate was significantly lower than the others: 24.8 versus 36.2% (*p* = 0.001).

**Discussion:**

Male sex, age older than 65 years, surgical time longer than 90 min and delayed drainage removal are risk factors for severe HO. Patients with one or more of those risk factors should be identified as good candidates for HO prophylaxis.

## Introduction

Total hip arthroplasty (THA), described as “the operation of the century” [1] for its good long term results, has a worldwide increasing diffusion [2, 3]. Heterotopic ossification (HO) [4], an abnormal formation of bone in extra-skeletal soft tissue [5, 6] is a common complication of this procedure and may cause significant functional limitations. HO is usually classified according to the Brooker classification system [7] and a recent meta-analysis described an average rate of HO in THA of 30% [8], while other works described rates between 15 and 90% [9, 10].

Several risk factors for HO after a THA are described in the literature. Among these, there are patient related factors (male gender, age, ankylosed hip, ankylosing spondylitis) [8, 11] and surgery related factors (surgical approach, blood loss, procedure duration and use of drainage) [7, 8, 12]. Currently, there are no orthopedic guidelines available on this topic [10], but a recent Cochrane Library meta-analysis demonstrated an HO incidence reduction of 54–64% with an adequate prophylactic therapy [13]. However, an extended prophylaxis to all the patients that underwent a THA could lead to an increased number of adverse events like gastrointestinal bleeding or radiation-related malignant transformation [8, 11]. For this reasons, definition of high risk patients appears extremely important in order to identify patients which may benefit most from the prophylactic treatment.

Aim of the study is to identify if age, sex, type of postero-lateral approach (mini vs extended), surgical time and time from surgery to drainage removal were independent risk factors for HO formation after THA.

## Materials and methods

This retrospective study was conducted in a single large teaching hospital, study protocol was approved by the local committee (2018/2022), trial number 038.815 (final approval on 26/6/2019).

Patients were included if they underwent a THA with postero-lateral approach during a 15 years period. The exclusion criteria were absence of X-rays follow-up longer than 2 years, HO prophylaxis protocol adoption or simultaneous bilateral THA.

The following data were collected: age at surgery, sex, type of approach (classical or minimally invasive), surgical time, time to drainage removal. Cut-off values were determined: 65 years for age, 90 min for surgery duration and 24 h for postoperative drainage removal.

Two orthopedic surgeons reviewed the two-year follow-up X-rays and classified the HO (if present) according to the Brooker classification system [14]: grade 0: no ossification; grade 1: islands of bone in the soft tissues around the hip; grade 2: bone spurs from the proximal femur or from the pelvis with at least 1 cm between opposing bone surfaces; grade 3: bone spurs from the proximal femur or from the pelvis with less than 1 cm between opposing bone surfaces and grade 4: apparent bone ankyloses of the hip. According to Eggli [15] and according to our experience patients’ satisfaction after THA significantly dropped from grade 2 HO, as well as walking capacity and use of analgesics. So HO was defined as “severe” if classified as major than grade 2. All data were analyzed with standard descriptive statistics. Univariate analysis was performed with regard to severe HO (yes or no). This was done with the chi-squared test or Fisher’s exact test for categorical outcomes and Student’s *t* test or the Mann–Whitney test for continuous outcomes. The Kolmogorov–Smirnov test was used to determine whether data were normally distributed. The relationship between severe HO and study characteristics was assessed with multivariate linear regression models. *p* values lower than 0.05 were considered statistically significant. All analyses were performed using Stata version 12 (Stata Corporation, College Station, TX, USA).

## Results

During the study period, 1859 patients underwent a THA with postero-lateral approach. In 589 cases no x-rays follow-up longer than 2 years was retrievable, 27 underwent an HO prophylaxis and 18 underwent a simultaneous bilateral THA, therefore 1225 patients were included in our study.

Mean age of 63.8 years (SD 13), 721 were women (58.9%) and 504 men (41.1%).

HO were found in 67.6% of cases. According to the Brooker classification system, 32.3% of patients were classified as grade 1, 16.5% as grade 2, 11.4% as grade 3 and 7.4% as grade 4 (Table 1). Men showed severe HO in 44.1% and women in 29.1%: the difference was statistically significant (*p* = 0.001).

**Table 1:** Distribution of heterotopic ossification according to Brooker classification

Brooker grade	Frequency	Rate (%)	Cumulative
0	397	3240	3242
1	396	3233	6475
2	202	1649	8124
3	140	1141	9264
4	90	737	100
Total	1225	100	

Among the 1225 patients, 48.6% of them was 65 years old or younger, while 51.4% was older. A significant difference in severe HO prevalence (*p* = 0.002) was found between the first and the second group, respectively with a rate of 30.3% and 39.9%.

In 75.4% of cases a classic postero-lateral approach was performed, while in 24.6% of cases a minimally invasive approach was used. Patients treated with the classic postero-lateral approach showed severe HO rate of 32.8% versus 27.1% found in those treated with the minimally invasive approach (*p* = 0.067).

Regarding surgical time, in 75.6% of cases surgery lasted 90 min or less, while in 24.4% of cases it lasted longer. In the first group the severe HO rate was 29.1%, while in the second group it was 35.7%, with a statistically significant difference (*p* = 0.033).

In 47.4% of patients the drainage was removed before 24 h from surgery, in 52.6% after 24 h. In the second group severe HO rate was significantly higher: respectively 24.8 versus 36.2% (*p* = 0.001).

Male sex, age older than 65 years, surgical time longer than 90 min and drainage removal after 24 h were significantly associated with higher rated of severe HO in the multivariable analysis (Table 2).

**Table 2:** Multivariate logistic regression between severe heterotopic ossification and male sex, age older than 65 years, surgery duration greater than 90 min and drainage removal after 2 or more post-operative day

Risk factors	Coef.	Std. err.	*P*	95% Conf. interval
Male sex	0.7041	0.1301	0.001	0.4491–0.9591
Age > 65 year	0.0241	0.0048	0.001	0.0146–0.0336
Surgery duration > 90 min	0.3398	0.1480	0.022	0.0497–0.6299
Drainage removal ≥ 2nd post-op day	0.249	0.1085	0.022	0.0363–0.4616

## Discussion

Our study demonstrates that male sex, age older than 65 years, surgical time longer than 90 min and delayed drainage removal are risk factor for severe HO development. Currently there is no consensus about the indications for HO prophylaxis (radiotherapy or nonsteroidal anti-inflammatory drugs (NSAIDs)) after THA [10, 16]. Radiotherapy has the best prophylaxis effectiveness for HO after THA but presents higher cost and complex management: for these reasons may be the most appropriate approach in high-risk patients (ipsilateral high grade HO) or in those with contraindications to NSAIDS [16–19]. Nowadays NSAIDs are the most commonly used prophylaxis [16, 20]: in particular selective cyclooxygenase 2 (COX-2) inhibitors were found to maintain equally effectiveness in preventing HO formation after hip surgery with a reduced gastrointestinal side effect compared with non-selective NSAIDs [18, 19].

In our study we found an overall HO incidence of 67.6% similarly to other authors who reported an incidence up to 90% [10, 21]. In contrast, a recent meta-analysis presented an average HO incidence after THA of 30% [8]: our higher incidence may be related to the inclusion criteria (patients were excluded if they received a HO prophylaxis) and, according to literature, prophylaxis reduces HO incidence of 54%-64% [13, 22].

The presence of HO has an important impact on the clinical outcome in total hip replacement. Literature reported that 15% of HO patients suffers from pain and limited range of motion of the involved hip [23]. Eggli et al. [15] showed that patients rate with mild or severe pain increased from less than 10% to more than 50% from lower to higher Booker grade. Moreover, is reported that walking capacity decrease and use of analgesic increase starting from Brooker grade 2 to higher grade. Also patient satisfaction was significantly influenced by HO degree, dropping from almost 90% in Brooker grade 0 to less than 30% in Brooker grade 3 and 4, with a significant difference in rating already between the patients in grade 0 and grade 2 [15].

In our study, a statistically significant correlation between male sex and severe HO was demonstrated and this result is consistent with the literature: male sex is widely considered a strong risk factor for clinical relevant HO after THA implant [8, 24, 25].

About the age, there is not a complete concordance between authors: Zhu et al. [8] and Eggli et al. [24] underlined that age is not a risk factor for HO in THA while Biz [22] showed a correlation between young age and HO formation. Furthermore, other authors considered age as a risk factor for HO [7, 11, 26] and our study support those statements.

The degree of soft tissue trauma during surgery and surgery duration are demonstrated to be important risk factors for HO development [7, 27, 28]. Hürlimann [29] reported that surgical approach significantly influences rate and degree of HO after THA and demonstrated a lower incidence of HO in minimally-invasive approaches, while Edwards [27] reported a statistically significant correlation between incisions longer than 10 cm or surgery duration longer than 60 min and higher grade of HO. In our study, we included only THA implanted with postero-lateral approach, divided between classical and mini-invasive. According to literature [27, 29], a higher prevalence of high grade HO in classical approach should be observed because of the wider soft tissue trauma: the results found was consistent with literature but without a statistical significance.

Surgical time is also considered as an indirect index of tissue damage: a long lasting surgery is usually due to a more complex treatment which implies a wider tissue trauma. In fact, we found a statistically significant higher rate in HO ≥ 2 between the patients that underwent surgery longer than 90 min.

To the best of our knowledge, just only one study focused on the intra-articular drainage as a potential risk factor: Di Benedetto [12] analyzed HO incidence in 425 THA, mostly performed with direct anterior approach, with and without drainage positioning. They showed a significantly higher HO rate in THA with drainage [12]. Similarly, our work showed an increased severe HO rate in patients with a prolonged drainage maintenance. The drainage may stimulate the HO starting process in different ways. Drainage positioning may be considered an additional source of tissue trauma and its presence may spread osteogenic mediators through soft tissues. It may also favor a locally recall of inflammatory factors acting as foreign body [12]. Moreover, the positioning and the prolonged maintaining of drainage could be considered an index of soft tissue trauma during surgery: a greater tissue trauma implies a greater bleeding and consequently the need of drainage positioning and its prolonged maintenance.

Our study presents several limits. Its retrospective single center nature may limit our conclusions. Furthermore, we used the Brooker classification system and the latter is based on a single anteroposterior X-ray of the pelvis and may under or overestimate the HO extension but it is the most widely diffused [7, 30]. Eventually, clinical evaluation was not performed therefore further clinical study should confirm is the proposed factors are also valid for clinical impairment related to HO.

Male sex, age older than 65 years, surgical time longer than 90 min and delayed drainage removal are risk factor for severe HO development. Patients with one or more of those risk factors should be identified as good candidates for HO prophylactic treatment.

## Data Availability

All data have been store in the dedicated repository of University of Turin.
